# Die traumatische TFCC-Läsion im Kindes- und Jugendalter – eine bisher vernachlässigte Verletzung?

**DOI:** 10.1007/s00113-025-01563-0

**Published:** 2025-04-11

**Authors:** Kristofer Wintges, Dirk Sommerfeldt, Hauke Rüther

**Affiliations:** 1 Klinik und Poliklinik für Kinderchirurgie, Universitätsklinikum Hamburg-Eppendorf, Martinistr. 52, 20246 Hamburg, Deutschland; 2 Abteilung für Kinder- und Jugendtraumatologie, Altonaer Kinderkrankenhaus gGmbH, Bleickenallee 38, 22763 Hamburg, Deutschland; 3 Klinik für Unfallchirurgie, Orthopädie und Plastische Chirurgie, Universitätsmedizin Göttingen, Robert-Koch-Straße 40, 37075 Göttingen, Deutschland

**Keywords:** Triangulärer fibrokartilaginärer Komplex, DRUG-Instabilität, Chronische Handgelenksschmerzen, Handgelenkarthroskopie, Handgelenksverletzung, Triangular fibrocartilage complex, Distal radioulnar joint instability, Chronic Wrist pain, Wrist arthroscopy, Wrist trauma

## Abstract

Verletzungen des triangulären fibrokartilaginären Komplexes (TFCC) kommen im Kindes- und Jugendalter zwar seltener vor als bei Erwachsenen, können jedoch für chronische Schmerzen sowie eine Instabilität im distalen Radioulnargelenk (DRUG) ursächlich sein, mit dem langfristigen Risiko einer Arthrose. Eine dislozierte distale Radiusfraktur mit Abriss des Processus styloideus ulnae nach einem Hochenergietrauma stellt dabei einen Risikofaktor für eine TFCC-Verletzung dar. Die Diagnostik umfasst eine gründliche klinische Untersuchung sowie bildgebende Verfahren wie Röntgen und Magnetresonanztomographie. Bei fehlender Instabilität kann eine konservative Therapie in den meisten Fällen erfolgreich sein. Besteht jedoch eine Instabilität oder zeigen sich nach 3 Monaten keine Besserungen unter konservativer Therapie, ist eine diagnostische Arthroskopie des Handgelenkes zu weiterer Diagnostik und gleichzeitigen Therapie indiziert. Hierbei können je nach Alter und Ausmaß der Verletzung verschiedene operative Techniken wie die transkapsuläre oder transossäre Refixation zum Einsatz kommen. Eine frühzeitige Diagnosestellung und Therapie sind entscheidend, um eine Schmerzfreiheit, ein stabiles DRUG und eine Rückkehr zu sportlichen Aktivitäten zu ermöglichen. Im eigenen Patientenkollektiv zeigen sich so bei 12 Kindern und Jugendlichen sehr gute Ergebnisse ohne größere Komplikationen.

## Abstract (en)

Although injuries to the triangular fibrocartilage complex (TFCC) are less common in children and adolescents than in adults, can lead to chronic pain and instability in the distal radioulnar joint (DRUJ), ultimately predisposing individuals to long-term osteoarthritis. A dislocated distal radius fracture with avulsion of the styloid process of the ulna following high-energy trauma is a risk factor for a TFCC injury. The diagnostics include a thorough clinical examination and imaging procedures, such as X‑ray and magnetic resonance imaging (MRI). If there is no instability of the DRUJ, conservative treatment can be successful in most cases. However, if there is instability of the DRUJ or if there is no improvement after 3 months of conservative treatment, diagnostic arthroscopy of the wrist is indicated for further diagnostics and concurrent treatment. Depending on the age and extent of the injury, various surgical techniques can be used, such as transcapsular or transosseous refixation. Early diagnosis and treatment are essential to achieve freedom from pain, a stable DRUJ and enable a return to sports activities. In our own patient collective 12 children and adolescents showed very good results with no major complications.

Obwohl das Wissen über den dreieckigen Faserknorpelkomplex (triangulärer fibrokartilaginärer Komplex, TFCC) in Bezug auf Anatomie, Funktion, Pathologie und Behandlungsmethoden beim Erwachsenen in den letzten Jahren erheblich zugenommen hat, wird die TFCC-Läsion bei Kindern und Jugendlichen immer noch vernachlässigt [1, 2].

## Anatomie, Epidemiologie und Inzidenz

**Abb. 1:**
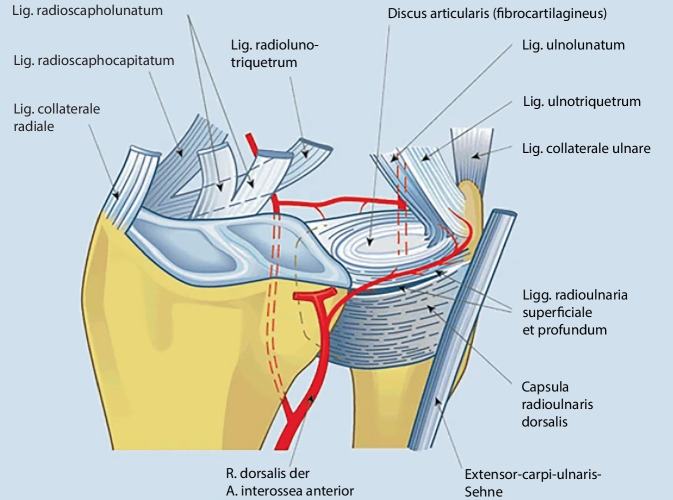
Aufbau des triangulären fibrokartilaginären Komplexes, seine Blutversorgung und die Beziehung zu den radialen Bandstrukturen. (Aus Schöll et al. [63]; mit freundlicher Genehmigung © Springer)

Der TFCC setzt sich aus dem Discus triangularis, den ulnokarpalen, dorsalen und palmaren radioulnaren, ulnolunaren und ulnotriquetralen Bändern sowie der Sehnenscheide des M. extensor carpi ulnaris zusammen (Abb. 1; [3]). Er fungiert zum einem als Kraftüberträger/Stoßdämpfer des Handgelenkes und gleichzeitig als Stabilisator des distalen Radioulnargelenkes (DRUG) und des Ulnokarpalgelenkes. Seine Verletzung ist eine relativ häufige Begleitverletzung bei Erwachsenen mit 35–78 % [4], mit in der Folge akuter und chronischer Handgelenkschmerzen, die zu einer eingeschränkten Beweglichkeit führen. Oft ist dies begleitet von einer Instabilität des DRUG [5]. Eine traumatische Verletzung des TFCC im Kindes- und Jugendalter galt bisher als sehr seltene Verletzung, wie Schachinger und Farr in ihrem Review zeigen konnten [2]. In den letzten Jahren wird diese Verletzung jedoch immer häufiger beobachtet. Dies könnte zum einen mit der verbesserten MRT-Diagnostik und der standardisierten und flächendeckenden Anwendung der Handgelenkarthroskopie, aber auch mit dem immer früheren Eintritt der Kinder und Jugendlichen in den Freizeit- und auch Leistungssport zusammenhängen [6, 7]. Dabei kommt es im modernen Jugendsport häufig zu Handgelenk- und Handverletzungen, auch in Sportarten, bei denen der Einsatz der Hand nicht im Vordergrund steht, wie z. B. Fußball, Skateboarden und Snowboarden [8–10]. Bei Kindern und Jugendlichen mit anhaltenden, posttraumatischen Schmerzen im ulnaren Handgelenk oder Instabilität wurde in 48,5–80,5 % der Fälle eine TFCC-Läsionen bei einer diagnostischen Handgelenkarthroskopie gefunden [5, 6, 11]. Während die TFCC-Läsion beim jungen Erwachsenen meist isoliert auftritt [12, 13], ist sie bei Kindern und Jugendlichen häufig mit einer dislozierten distalen Radiusfraktur (52–79 %) [1, 14] und dort v. a. mit SH-II-Frakturen (20 %) assoziiert [1]. Die Inzidenz von TFCC-Läsionen bei distalen Radiusfrakturen beträgt dabei laut Anderson et al. 2 % [1]. Betroffen sind meist Jugendliche im Alter von 12 bis 14 Jahren [1, 15].

## Diagnostik

### Anamnese und klinische Untersuchung

**Abb. 2:**
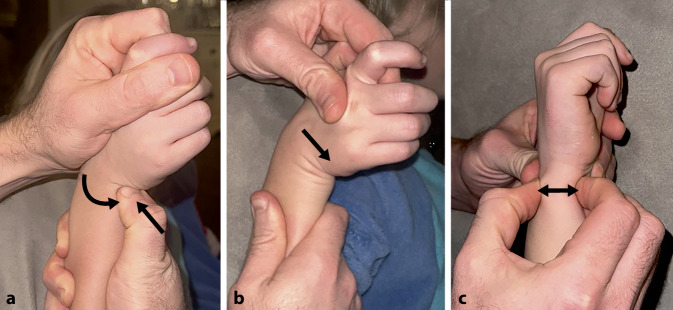
Durchführung 3 gängiger diagnostischer Tests zur Untersuchung des TFCC bzw. DRUG: **a** Fovea-Zeichen, **b** TFCC-Kompressionstest, **c** Ballottement-Test. Die *Pfeile* visualisieren die Richtung der manuellen Druckausübung oder Bewegung, die während des jeweiligen Tests ausgeführt wird

Die Diagnosestellung einer TFCC-Verletzung kann aufgrund unspezifischer Symptome häufig eine Herausforderung darstellen. Kinder und Jugendliche mit einer traumatischen TFCC-Verletzung klagen meistens über Schmerzen sowie ein Instabilitätsgefühl auf der ulnaren Seite des Handgelenks. Häufig manifestieren sich diese Beschwerden bereits nach der Gipsabnahme nach einer operativ oder konservativ therapierten distalen Radiusfraktur. Die Symptome verschlimmern sich meist bei Ulnardeviation des Handgelenks, Drehbewegungen, Kraftgriffen und axialer Kompression oder Gewichtsbelastung, wie z. B. Liegestützen. Das zuverlässigste klinische Zeichen für einen ulnarseitigen peripheren TFCC-Riss ist das ulnare Fovea-Zeichen (Abb. 2a). Dabei kann der Untersucher einen punktförmigen Schmerz an der ulnaren Kapsel palmar der ECU-Sehne auslösen. Dieser wird durch eine passive Rotation des Unterarms noch verstärkt und kann mit einem „Klicken“ einhergehen. Die Sensitivität und Spezifität liegen laut Tay et al. bei 95,2 % bzw. 86,5 % [16]. Ein weiterer Test zur Untersuchung des TFCC ist der TFCC-Kompressionstest, wobei das Handgelenk unter axialer Kompression in Ulnardeviation gebracht wird (Abb. 2b). Ein positives Testergebnis liegt vor, wenn ein Klicken oder Krepitieren mit Schmerzen einhergeht [17]. Ein einfacher und zuverlässiger Test zur Beurteilung der DRUG-Stabilität ist zudem der Ballottement-Test (Abb. 2c; [18]). Dieser Test besteht aus einer passiven anteroposterioren Translationsbewegung der Ulna zum Radius in neutraler Rotation, vollständiger Supination und Pronation. Eine abnorme Translation des Ulnakopfes deutet dabei auf eine vollständige TFCC-Ruptur hin. Je nach Ausmaß der Instabilität können die Symptome unauffällig oder stärker beeinträchtigend sein, daher wird eine Untersuchung im Vergleich zur kontralateralen Seite empfohlen [19].

#### Merke.

Patienten mit einer TFCC-Ruptur klagen typischerweise bereits nach der Gipsabnahme nach distaler Radiusfraktur über ulnarseitige Handgelenkbeschwerden.

### Röntgen und Magnetresonanztomographie

**Abb. 3:**
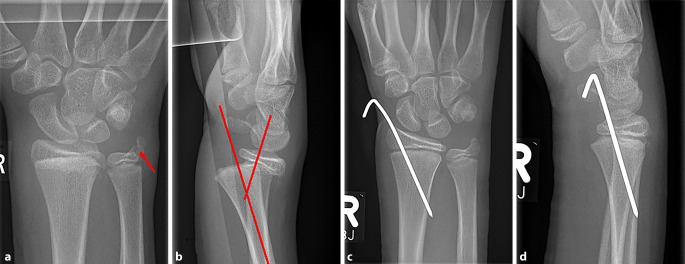
Ein 13-jähriger Junge erlitt während eines Fußballspiels einen Sturz, der zu einer distalen Radiusfraktur vom Typ II nach Salter und Harris führte. Dabei zeigten die Röntgenaufnahmen des Handgelenkes dp (**a**) und seitlich (**b**) einen basisnahen Abriss des PSU (*roter Pfeil*) und eine deutliche dorsale Dislokation der Epiphyse. Die Fraktur wurde geschlossen reponiert und mittels perkutaner Kirschner-Draht-Osteosynthese stabilisiert. Die postoperativen Röntgenaufnahmen des Handgelenkes dp (**c**) und seitlich (**d**) zeigten die anatomische Reposition des Radius sowie des PSU

Die Diagnostik bei Verdacht auf eine Verletzung des TFCC stellt weiterhin eine Herausforderung dar, da bis heute kein einziges bildgebendes Verfahren eine perfekte Sensitivität und Spezifität aufweist. Als Goldstandard gilt weiterhin die diagnostische Arthroskopie, die gleichzeitig die Möglichkeit einer therapeutischen Maßnahme bietet. Da es sich jedoch um ein invasives Verfahren handelt, wird sie nur bei starkem klinischen Verdacht eingesetzt [20]. Eine Röntgenaufnahme des Handgelenks bei Patienten mit Handgelenkschmerzen ist unerlässlich. Obwohl eine isolierte TFCC-Ruptur radiologisch schwer darstellbar ist, können Hinweise auf eine assoziierte DRUG-Instabilität wie eine distale Ulnaverschiebung, eine Verbreiterung des DRUG (> 5 mm) oder eine ulnare Styloidfraktur (Abb. 3a) gefunden werden. Letztere gilt dabei nicht mehr als sicherer Indikator für eine DRUG-Instabilität, sondern lediglich als Risikofaktor, unabhängig von der Fragmentgröße und -verschiebung sowie Frakturlokalisation [21–23]. Darüber hinaus führt zumindest beim Erwachsenen eine dorsale Angulation der begleitenden oder vorgelegenen distalen Radiusfraktur von 20–30° mit dorsaler Verschiebung (Abb. 3b) häufig zu einer begleitenden TFCC-Ruptur, wie biomechanische Studien von Scheer und Adolfsson zeigen konnten [24]. Zudem muss eine Fehlstellung des Radius ausgeschlossenen werden, da gerade eine Translation zu einer Instabilität im DRUG führen kann.

#### Merke.

Eine dislozierte distale Radiusfraktur mit begleitendem PSU-Abriss nach einem Hochenergietrauma stellt einen signifikanten Risikofaktor für eine TFCC-Läsion dar.

**Abb. 4:**
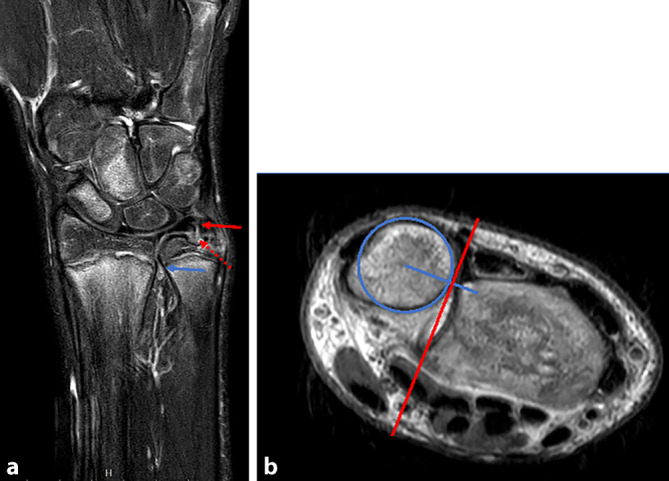
Magnetresonanztomographie des Handgelenks des Patienten aus Abb. 1 nach 10 Wochen bei anhaltenden ulnaren Handgelenkschmerzen und Instabilitätsgefühl. In der koronaren T2-gewichteten Aufnahme (**a**) zeigen sich eine Ruptur des oberflächlichen Anteils (*roter Pfeil*) sowie ein fovealer Ausriss des tiefen Anteils (*roter gestrichelter Pfeil*) des ulnaren TFCC mit begleitender Flüssigkeitsansammlung im DRUG (*blauer Pfeil*). Die axiale Aufnahme (**b**) visualisiert eine Subluxation des DRUG, die durch die Berechnung der radioulnaren Ratio quantifiziert wird [29]

Die MRT ist die am häufigsten verwendete Methode zur Beurteilung des TFCC (Abb. 4a). Durch Fortschritte bei der Feldstärke, der Spulentechnologie und der Impulsabfolge konnte die diagnostische Leistung deutlich verbessert werden. Trotzdem weist das MRT weiterhin nur eine Sensitivität von 71–100 % bzw. Spezifität von 82–86 % beim Nachweis einer TFCC-Verletzung auf [25, 26]. Das Vorliegen eines Ergusses im DRUG kann ein indirekter Hinweis auf eine Verletzung des TFCC, auch bei fehlendem Nachweis einer TFCC-Läsion, sein (Abb. 4a; [27]). Eine zusätzlich bestehende Subluxation des DRUG – Ulnakopf gegenüber Sigmakerbe des Radius – ist ein weiterer Hinweis für eine foveale TFCC-Ruptur (Abb. 4b; [28]). Hierzu stehen verschiedene Messparameter mit unterschiedlicher Genauigkeit zur Verfügung [29]. Durch die Ergänzung der MRT um eine zusätzliche Arthrographie lässt sich die Sensitivität auf 91,7–95,8 % und die Spezifität auf 85–94 % erheblich verbessern. Diese verbesserte Genauigkeit hat damit das Potenzial, die diagnostische Arthroskopie zu ersetzen [20, 26, 30]. Dieses Verfahren ist jedoch auch invasiv und erfordert in der Regel eine Sedierung. Insbesondere periphere TFCC-Läsionen, die überwiegend bei Kindern und Jugendlichen auftreten, werden jedoch weiterhin mit einer deutlich niedrigeren Sensitivität als zentrale Risse diagnostiziert, was auf den partiellen Volumeneffekt der radioulnaren Bänder zum PSU in der Standardarthrographie zurückzuführen ist [31].

#### Merke.

Die diagnostische Arthroskopie ist der Goldstandard zur Detektion und zur genauen Klassifikation einer TFCC-Läsion.

## Klassifikation

Die TFCC-Läsionen werden traditionell nach der Klassifikation von Palmer (Tab. 1; [3]) eingeteilt, wobei zwischen „traumatischen“ (*Klasse 1*) und „atraumatischen“ Ursachen (*Klasse 2*) unterschieden wird. Dieser Ansatz ist jedoch durch mehrere Aspekte eingeschränkt. Erstens ist es nicht immer möglich, zwischen traumatischen und vorbestehenden degenerativen Läsionen zu unterscheiden. Zweitens ist die Grenze zwischen zentralen und peripheren Läsionen nicht klar definiert. Schließlich und v. a. wird nicht zwischen der tiefen und der oberflächlichen Schicht des ulnaren TFCC unterschieden. Mit Ausnahme der seltenen Läsion der ulnokarpalen Bänder (*Typ 1C nach Palmer*) werden alle peripheren Läsionen als Typ 1B nach Palmer klassifiziert, obwohl sie unterschiedliche therapeutische Ansätze erfordern. Im Jahr 2009 führte Atzei [19] daher ein neues Klassifikationssystem ein, das insbesondere die Anatomie des ulnaren TFCC und die Behandlungsmöglichkeiten der verschiedenen Läsionen berücksichtigt (Tab. 1; [32 33]). Das Fehlen einer begleitenden Fraktur des PSU wurde im Verlauf durch Atzei und Luchetti ergänzt [34]. Trotz der weit verbreiteten Palmer- und Atzei-Klassifikationen bleiben zahlreiche, weniger häufige und v. a. periphere TFCC-Läsionen unberücksichtigt. Um eine umfassende, klinisch relevante und einfach anwendbare Klassifikation zu schaffen, entwickelten Schmitt et al. die „CUP“-Klassifikation (Tab. 1). Diese unterteilt TFCC-Läsionen in die Kategorien „zentral“, „ulnar“ und „peripher“, um eine präzise Grundlage für die Therapieplanung zu bieten [35]. Dennoch wird in den vorliegenden Fallberichten nach wie vor überwiegend die Palmer-Klassifikation verwendet, wobei bei Jugendlichen vorwiegend Verletzungen der Typen 1B (54,1 %) und 1D (15,4 %) nach Palmer vorliegen [2].

**Tab. 1:** Klassifikationen der nicht-knöchernen TFCC-Läsionen der häufigsten verwendeten Klassifikationen sowie deren Therapieempfehlungen. (Modifiziert nach Schmitt et al. [36])

Typ der TFCC-Läsion	CUP-Klassifikation^**^	Palmer-Klassifikation	Atzei-Klassifikation	Therapieempfehlungen
Mukoide Diskusdegeneration	C1	IIA, IIB	4	Abwarten und beobachten
Diskusperforation < 3 mm	C2	IA	4	Débridement
Diskusperforation > 3 mm*	C3	ID, IIC–IIE	4	Débridement und Ulnaverkürzungsosteotomie
Riss der oberflächlichen Anheftung am PSU	U1	IB	1	Ruhigstellung oder Refixation
Riss der tiefen Anheftung am PSU	U2	IB	3	Refixation oder Naht
Komplettruptur der Anheftung am PSU	U3	IB	2	Refixation oder Naht
Riss des Meniskushomologs und/oder der ulnokarpalen Kapsel	P1	–	–	Ruhigstellung oder Refixation
Riss der ulnokarpalen Bänder (ulnolunat, ulnotriquetral)	P2	IC	–	Ruhigstellung oder Refixation
Riss der radioulnaren Bänder (Radial- oder Mittelsegmente)	P3	–	–	Refixation

^*^Atypische Verletzungen eingeschlossen

^**^Zusätzliche knöcherne Verletzungen werden mit # *gekennzeichnet*

*C* *=* *zentral*, *U* *=* *ulnar*, *P* *=* *peripher*, *I* *=* *traumatisch*, *II* *=* *degenerativ*,

## Therapie

### Konservative Therapie

Konservative Maßnahmen wie die Anwendung nichtsteroidaler Antirheumatika (NSAR), Ruhigstellung mittels Gips oder Orthese für 4 bis 6 Wochen sowie Ergotherapie können bei Patienten mit einer Läsion des TFCC ohne DRUG-Instabilität zu einer Beschwerdelinderung führen [37, 38]. Park et al. konnten in ihrer Studie mit einer Unterarmimmobilisation eine Ausheilung von 57 % erreichen [38], welche durch eine Ruhigstellung über das Ellenbogengelenk hinaus sogar auf 75 % gesteigert werden konnte [39]. Eine Orthese (WristWidget®, Hawaii, USA) hat sich bei Erwachsenen v. a. mit stabilem DRUG und degenerativen TFCC-Läsionen als vielversprechend erwiesen (Abb. 6a und b; [40]). Für Kinder und Jugendliche liegen bislang jedoch keine Studiendaten vor.

Bei ausbleibender Besserung kann ggf. eine zusätzliche Kortikosteroidinjektion zur Behandlung der meist begleitenden Synovialitis erwogen werden [15, 41]. Daten zu Kindern und Jugendlichen liegen hier jedoch nicht vor, sodass es sich um eine Off-label-Behandlung handelt; diese sollte dringend aufgeklärt werden. Studien bei Erwachsenen zeigten, dass bei etwa 60 % der Patienten mit einer TFCC-Läsion nach konservativer Therapie eine Besserung der Symptome eintrat [34, 38, 42]. Auch bei Patienten mit zentralen TFCC-Rissen können konservative Maßnahmen trotz des begrenzten Heilungspotenzials in einigen Fällen zu einer Beschwerdefreiheit führen. Bei einer kompletten TFCC-Ruptur mit Instabilität des DRUG ist das Ergebnis einer konservativen Therapie in der Regel schlechter [39]. Bleibt diese Instabilität langfristig bestehen, führt dies zu einer Schädigung des Knorpels mit dem Endstadium einer degenerativen Arthrose im DRUG oder im Radiokarpalgelenk [14, 43, 44].

#### Merke.

Ein instabiles DRUG bei bzw. nach distaler Radiusfraktur gilt als behandlungsbedürftig.

### Operative Therapie

Fischer et al. empfehlen daher bei Kindern mit anhaltenden ulnaren Handgelenkschmerzen nach einem Trauma eine frühzeitige diagnostische Arthroskopie, um Verzögerungen und unbefriedigende konservative Behandlungen zu vermeiden [45]. Dies ist besonders wichtig, da die besten Heilungschancen innerhalb der ersten 3 Monate vorliegen [34] und eine verspätete operative Versorgung oft aufwendigere Eingriffe [1] oder Reeingriffe nach sich zieht [46].

Indikationen zur Operation:

- persistierende Beschwerden trotz konservativer Therapie > 3 Monate [46],
- DRUG-Instabilität („floating PSU“) oder Subluxation/Luxation im MRT,
- begleitende intraartikuläre Verletzungen wie z. B. Knorpelschäden.

**Abb. 5:**
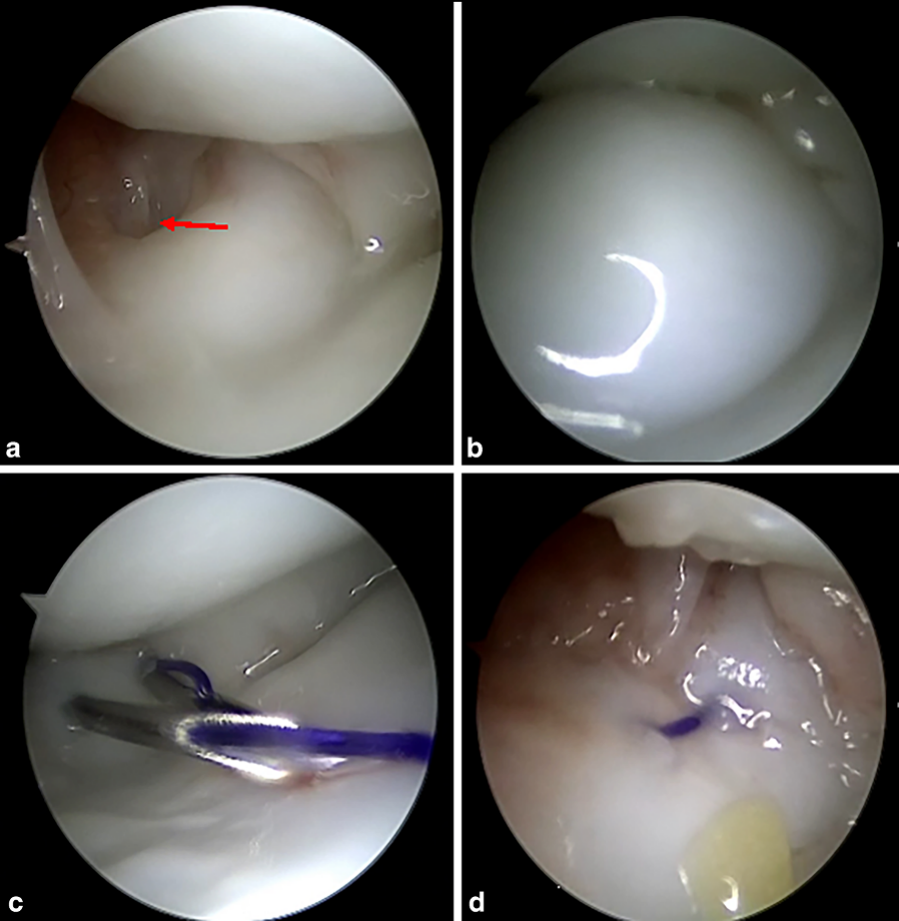
Die diagnostische Arthroskopie (trocken) über das 3/4-Portal des Handgelenks des Patienten aus Abb. 1 und 2 zeigt eine Ruptur des oberflächlichen Anteils des ulnaren TFCC (*roter Pfeil*, **a**). Der Hook-Test (**b**) erbringt zudem den Nachweis des fovealen Abrisses des TFCC durch Abheben des gesamten TFCC-Komplexes. Die arthroskopische Therapie erfolgte mittels transkapsulärer Refixation in Outside-in-Technik unter Verwendung von 2 Kanülen und vorgelegten PDS-Fäden bei noch offenen Wachstumsfugen (**c,** **d**)

Aktuelle Studien zeigen, dass sowohl die offene als auch die arthroskopische Versorgung von TFCC-Läsionen zu einer vergleichbaren Schmerzreduktion und Wiederherstellung der Handgelenkfunktion führen [47–49]. Die arthroskopische Technik, die sowohl trocken als auch feucht durchgeführt werden kann, bietet jedoch den Vorteil einer geringeren Invasivität, einer schnelleren Rehabilitation und einer besseren intraoperativen Übersicht zur Beurteilung von Begleitpathologien (Abb. 5a). Während der Untersuchung können zudem die Spannung und auch die Belastbarkeit des TFCC mittels Hook- [50] und Trampolintest [51] bewertet und die Ruptur besser klassifiziert werden (Abb. 5b). Anschließend können ulnarseitige oder zentrale Risse (*Typen 1B und 1D nach Palmer*) arthroskopisch genäht oder transossär refixiert und radiale oder zentrale Risse (*Typen 1A, 1C und 1D nach Palmer*) débridiert werden [14, 15, 46, 52–56]. Die Wahl der geeigneten Technik hängt von der Art und Ausdehnung der Läsion sowie vom Alter des Patienten ab. Bei Kindern mit noch offener Wachstumsfuge der Ulna ist die transkapsuläre Refixation mittels „Inside-out“- oder „Outside-in“-Nähten eine bewährte Methode [53, 54, 57]. Diese Technik ermöglicht eine sichere Fixation des TFCC und Stabilisierung des DRUG ohne Schädigung der Wachstumsfuge (Abb. 5c, d). Hierbei sollte besonders darauf geachtet werden, den dorsalen sensiblen Hautast des N. ulnaris zu schonen und die Nahtknoten tief genug zu versenken, um Hautirritationen oder Wundheilungsstörungen zur vermeiden [58, 59]. Bei nahezu geschlossenen oder geschlossenen Wachstumsfugen sollte bei kompletter TFCC-Ruptur eine arthroskopisch gestützte bzw. offene foveale transossäre Refixation oder Refixation mittels Fadenanker oder knotenlosem System erfolgen [56, 60, 61], da diese Verfahren zu einer noch höheren Stabilität im Vergleich zur transkapsulären Refixation führen [62].

#### Merke.

Die Therapie einer TFCC-Ruptur bei Kindern und Jugendlichen sollte zeitnah in einem geeigneten Zentrum erfolgen.

### Postoperative Nachbehandlung

**Abb. 6:**
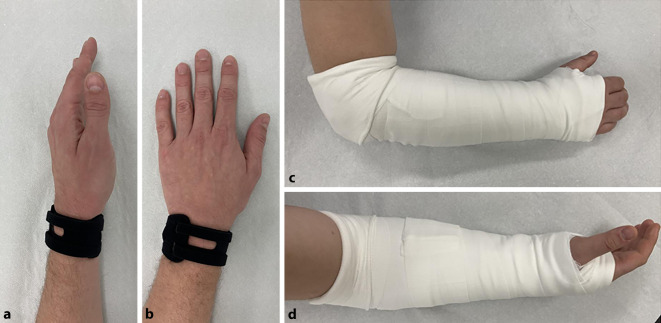
Zur Behandlung von TFCC-Rupturen stehen verschiedene Ruhigstellungsmöglichkeiten zur Verfügung. Hierzu zählen die WristWidget®-Orthese für eine konservative Therapie (**a**, **b**) sowie die „Sugar-tong“-Gipsschiene zur postoperativen Immobilisierung nach einer Refixation oder Naht des TFCC (**c**, **d**)

Die Nachbehandlung richtet sich nach der vorangegangenen Therapie. Patienten, bei denen eine Naht oder Refixation des TFCC erfolgt ist, empfehlen die Autoren postoperativ die Ruhigstellung im Oberarm-Cast für 4 bis 6 Wochen mit anschließendem Wechsel auf eine Handgelenkorthese für weitere 2 bis 4 Wochen [15]. Beschriebene Alternativen sind die Ruhigstellung für 2 Wochen mittels „Sugar-tong“(Zuckerzangen)-Gipsschiene und Wechsel auf einen Münster-Gips für weitere 4 Wochen (Abb. 6c und d; [53]) bzw. die Ruhigstellung für 2 bzw. 4 Wochen im Oberarm-Cast mit Wechsel für weitere 2 bzw. 4 Wochen auf einen Unterarm-Cast [46, 54]. Bei Patienten, die ausschließlich ein Débridement des TFCC erhalten haben, ist in der Regel eine Ruhigstellung für maximal 2 Wochen notwendig. Die ergotherapeutische Behandlung sollte mit der Abnahme des Gipses beginnen. Ab der 8. Woche können gezielte Kräftigungsübungen und propriozeptives Training in den Therapieplan integriert werden. Die Wiederaufnahme sportlicher Aktivitäten ist individuell unterschiedlich und hängt vom Heilungsverlauf ab. In der Regel ist eine Rückkehr zum Sport nach 3 bis 4 Monaten möglich.

## Ergebnisse

Im Zeitraum von 2019 bis 2024 wurden 12 Kinder und Jugendliche im Alter von 9 bis 16 Jahren (mittleres Alter von 13,2 Jahre) aufgrund anhaltender Schmerzen im ulnaren Handgelenk bzw. einer bestehenden Instabilität behandelt (Tab. 2). Bei allen Patienten wurde im MRT nach klinischem Verdacht eine Ruptur des TFCC diagnostiziert.

**Tab. 2:** Demografische Daten der behandelten Patienten, Ausmaße der erlittenen Verletzungen an distalem Radius, Ulna und TFCC sowie durchgeführte Therapien

Geschlecht	Alter	Unfallart	Radiusfraktur	PSU-Fraktur	CUP-Klassifikation	Therapie
M	13 Jahre	Fußball	Typ SH II	Basis	U2#	Transossäre Refixation
M	16 Jahre	Basketball	Typ SH II	Spitze	U1#	Transkapsuläre Naht
M	13 Jahre	Fußball	Typ SH I	Basis	C2U3#	Transossäre Refixation + Débridement
M	13 Jahre	Fußball	Typ SH I	Basis	U3#	Transossäre Refixation
M	14 Jahre	Fußball	Typ SH I	Basis	U2#	Transossäre Refixation
M	15 Jahre	Ju-Jutsu	Intraartikuläre Fraktur	Basis	U2#	Transossäre Refixation
W	15 Jahre	Volleyball	Metaphysäre Wulstfraktur	Basis	U3#	Transkapsuläre Naht
M	16 Jahre	Fußball	Typ SH II	Basis	U3#	Transossäre Refixation
M	11 Jahre	Sturz vom Baum	Typ SH II	Basis	C2U2#	Transkapsuläre Naht + Débridement
M	9 Jahre	Fußball	Typ SH II	Basis	U1#	Transkapsuläre Naht
M	13 Jahre	Eiskunstlauf	Diametaphysäre Fraktur	Basis	U3#	Transkapsuläre Naht
W	11 Jahre	Cheerleading	Metaphysäre Fraktur	Spitze	U3#	Transkapsuläre Naht

Die Patientenkohorte setzte sich aus 10 Jungen und 2 Mädchen zusammen. In allen Fällen war ein Sturzereignis, fast ausschließlich bei sportlicher Aktivität, die Ursache für die Verletzung. Die typischen Verletzungsmuster umfassten eine distale Radiusfraktur mit einem PSU-Abriss und einer begleitenden TFCC-Läsion. Dabei lag bei 41 % der Patienten eine SH-II-Fraktur des distalen Radius vor. Ein PSU-Abriss war in allen Fällen nachweisbar, wobei 2 der Patienten Spitzenabrisse und 10 Patienten basale Frakturen aufwiesen.

In allen Fällen fanden sich ulnare TFCC-Läsionen, die nach der CUP-Klassifikation klassifiziert wurden. Die operative Behandlung erfolgte entweder durch eine transossäre Refixierung (6 Fälle) oder transkapsuläre Naht (6 Fälle). In 2 Fällen lagen zusätzlich zentrale Einrisse im TFCC vor; diese wurden zusätzlich débridiert.

Nach einer durchschnittlichen Nachbeobachtungszeit von im Mittel 12 Monaten zeigte sich bei allen Patienten eine vollständige Wiederherstellung des Bewegungsumfangs (Range of Motion) im Vergleich zum gesunden Handgelenk. Sowohl klinisch als auch radiologisch gab es bei den transossär refixierten Patienten keine Hinweise auf eine Wachstumsstörung der distalen Ulna.

Als vorübergehende Komplikationen traten in einem Fall Parästhesien im Bereich des dorsalen Hautastes des N. ulnaris und in 2 Fällen Wundheilungsstörungen aufgrund von störendem Knotenmaterial auf. Letztere konnten durch Kürzen der Knoten in einer Revisionsoperation behoben werden.

Die Rückkehr zum Sport erfolgte nach 3 bis 4 Monaten. Alle Patienten konnten ihre ursprüngliche Sportart wieder aufnehmen.

## Fazit für die Praxis

- TFCC-Läsionen können im Kindes- und Jugendalter isoliert oder als Begleitverletzung von distalen Radiusfrakturen häufig ursächlich für einen persistenten ulnaren Handgelenkschmerz sein.
- Bei Verdacht auf eine TFCC-Läsion sind neben der klinischen Untersuchung des ulnokarpalen Handgelenkkomplexes eine MRT und ggf. eine Handgelenkarthroskopie zur definitiven Diagnosestellung unerlässlich.
- Die besten Heilungstendenzen bestehen innerhalb der ersten 3 Monate nach der Verletzung.
- Unbehandelte TFCC-Läsionen können zu einer Instabilität im DRUG, konsekutiv zu chronischen Schmerzen und langfristig zu einer Arthrose führen.
- Liegt eine Instabilität des DRUG vor, kann eine nicht korrekt rekonstruierte distale Radiusfraktur ursächlich hierfür sein.
- Komplette Abrisse sowie DRUG-Instabilitäten sollten in der Regel operativ versorgt werden.
- Die minimalinvasive Methode sollte, wenn möglich, bevorzugt werden.
- Zeitnah erkannt und adäquat therapiert, finden sich sehr gute Behandlungsergebnisse.

## Data Availability

Die in dieser Studie erhobenen Datensätze können auf begründete Anfrage beim Korrespondenzautor angefordert werden.
